# A Regularized Stochastic Block Model for the robust community detection in complex networks

**DOI:** 10.1038/s41598-019-49580-5

**Published:** 2019-09-13

**Authors:** Xiaoyan Lu, Boleslaw K. Szymanski

**Affiliations:** 1 Social and Cognitive Networks Academic Research Center and Department of Computer Science, Rensselaer Polytechnic Institute, Troy, NY 12180 USA; 2 Społeczna Akademia Nauk, Łódź, Poland

**Keywords:** Mathematics and computing, Physics

## Abstract

The stochastic block model is able to generate random graphs with different types of network partitions, ranging from the traditional assortative structures to the disassortative structures. Since the stochastic block model does not specify which mixing pattern is desired, the inference algorithms discover the locally most likely nodes’ partition, regardless of its type. Here we introduce a new model constraining nodes’ internal degree ratios in the objective function to guide the inference algorithms to converge to the desired type of structure in the observed network data. We show experimentally that given the regularized model, the inference algorithms, such as Markov chain Monte Carlo, reliably and quickly find the assortative or disassortative structure as directed by the value of a single parameter. In contrast, when the sought-after assortative community structure is not strong in the observed network, the traditional inference algorithms using the degree-corrected stochastic block model tend to converge to undesired disassortative partitions.

## Introduction

The study of modular structure in networks has a long history in the literature^1–4^. The primary focus of this line of work has been the discovery of the assortative community structures in which the network nodes are partitioned into communities with edges more numerous inside them than across them. In complex networks, the modular structures include not only such assortative community structures but also other mixing patterns, like the core-periphery structures^5^ and the bi-partite structures^6^. These various structures found in networks demand a more comprehensive class of community detection models than the assortative ones, targeted by the modularity-based approaches^2^. The modularity maximization is one of the most widely used approaches for assortative community detection. It aims at maximizing the modularity of the network partitions. The modularity is a broadly accepted quality metric that compares the number of edges observed in each community to the expected number of such edges in a random graph with the same degree sequence. As shown in^7^, the modularity maximization is, in fact, equivalent to the maximum likelihood estimation of the degree-corrected planted partition model which is a special case of the degree-corrected stochastic block model^8^. Therefore, the inference of network partition from the stochastic block model can be considered a more general approach to network clustering than the traditional modularity-based community detection algorithms. The former can discover a variety of network structures, different from the traditional assortative community structures, such as the disassortative core-periphery structures^5^.

The standard stochastic block model^9^ is a generative graph model which assumes the probability of connecting two nodes in a graph is determined entirely by their block assignments. In the following, we will denote the block assignment of any node *l* by *g*_*l*_. In the standard stochastic block model, the number of edges between nodes *i* and *j* follows the *Bernoulli* distribution with the mean *ω*_*gi*,*gj*_. Hence, this model is fully specified by the block assignments of nodes {*g*_*l*_} and the mixing matrix Ω = {*ω*_*rs*_} governing the probabilities of observing one edge between each pairs of nodes from blocks *r* and *s*. If the diagonal elements *ω*_*rr*_ in the mixing matrix are larger than the off-diagonal elements, then the networks generated by the stochastic block model have the traditional assortative communities. When the off-diagonal elements *ω*_*rs*_ for *r* ≠ *s* are larger than the diagonal elements, the generated network contains disassortative mixing patterns, such as the structures observed in the core-periphery graphs^5^ and in the bi-partite graphs^6^. In general, the inference using the stochastic block model aims at discovering Ω and {*g*_*l*_} which maximize the likelihood of generating the observed network. It does not impose any constraint on the assortativity of the mixing pattern Ω.

The standard stochastic block model assumes all nodes in a community have the same expected degree. However, a block in the realistic community structure often contains nodes with heterogeneous degrees. To address this issue, the degree-corrected stochastic block model^8^ extends the standard stochastic block model by defining the expected number of edges between nodes *i* and *j* as *λ*_*ij*_ = *ω*_*gi*,*gj*_*β*_*i*_*β*_*j*_. Here the parameter *λ*_*ij*_ represents the expected number of edges, which is important because multi-edges (multiple edges between pair of nodes) are allowed in the model^8^. The model parameter *β*_*l*_ is associated with each node *l*. The same notation *λ*_*i,j*_ is used for denoting probability of an edge between pair of nodes in the standard stochastic block model, so it is important to note that it has different meaning here. The expected node degrees in the degree-corrected stochastic block model, given the maximum likelihood estimates of these model parameters, are equal to the degrees observed in the input network. Hence, the degree-corrected stochastic block model allows a community to have a wide range of node degrees. This simple yet effective degree-correction modification improves the performance of statistical inference of community structures in complex networks.

As suggested in^10^, both the traditional assortative community structures and the disassortative structures are potentially good fits to the degree-corrected stochastic block models. Hence, depending on the starting point, inference algorithms, e.g., the Markov chain Monte Carlo, tend to converge to the local maxima of the likelihood, which may correspond to the disassortative structures. To address this problem, in this paper, we propose a regularized stochastic block model which provides an extra regularization term to guide the discovery of assortative or disassortative structure by the statistical inference using the stochastic block model. Unlike the modularity maximization algorithm which always attempts to find traditional assortative communities, the inference using this regularized stochastic block model controls with a single parameter the mixing patterns discovered in the given network.

## Results

### The degree-corrected stochastic block model

The degree-corrected stochastic block model^8^ is a generative model of graphs in which the edges are randomly placed between nodes. Let *A* be the adjacency matrix of an unweighted undirected multigraph, and let *A*_*ij*_ denote the number of edges between nodes *i* and *j* in this multigraph. The multi-edges and self-loop edges are practical in certain networks such as the World Wide Web where a web page may contain multiple hyperlinks pointing to other pages and to itself. Such edges are less common in social networks. However, most social networks are very sparse, so the impact of multi-edges and self-loop edges is negligible in such networks. For the convenience of notations, let a node *i* with *k* self-loop edges be represented by the diagonal adjacency matrix element *A*_*ii*_ = 2*k*.

The degree-corrected stochastic block model assumes the number of edges between two different nodes *i* and *j* follows the *Poisson* distribution with mean *λ*_*ij*_, while the number of self-loop edges at node *l* follows the *Poisson* distribution with mean $\frac{1}{2}{\lambda }_{ll}$. Given the parameters {*λ*_*ij*_}, the likelihood of generating *A* is

1 $$P(A|\{{\lambda }_{ij}\})=\prod _{i}\frac{{(\frac{1}{2}{\lambda }_{ii})}^{{A}_{ii}\mathrm{/2}}}{(\frac{1}{2}{A}_{ii})!}{e}^{-{\lambda }_{ii}\mathrm{/2}}\prod _{i < j}\frac{{\lambda }_{ij}^{{A}_{ij}}}{{A}_{ij}!}{e}^{-{\lambda }_{ij}}\mathrm{.}$$

Here, *λ*_*ij*_ defines the expected number of edges rather than the probability, because multi-edges are allowed in this model. In an unweighsted undirected network, after ignoring all terms independent of the *λ*_*ij*_, the corresponding log-likelihood simplifies to

2 $$\log \,P(A|\{{\lambda }_{ij}\})=\frac{1}{2}\sum _{ij}({A}_{ij}log{\lambda }_{ij}-{\lambda }_{ij}\mathrm{).}$$

In the degree-corrected stochastic block model, the model parameter *λ*_*ij*_ is defined as *λ*_*ij*_ = *ω*_*gi*,*gj*_*β*_*i*_*β*_*j*_ where for a node *l*, *β*_*l*_ denotes its parameter. Given the block assignments {*g*_*i*_}, the authors of ^8^ obtain the maximum likelihood estimates of the model parameters as ${\hat{\beta }}_{i}=\frac{{k}_{i}}{{\kappa }_{{g}_{i}}}$ and ${\hat{\omega }}_{rs}={m}_{rs}$, where *k*_*i*_ is the degree of node *i*, *κ*_*r*_ is the sum of the degrees of all nodes in a block *r*, and *m*_*rs*_ is the total number of edges between different blocks *r* and *s*, or, if *r* = *s*, twice the number of edges in the block *r*.

### Assortative and disassortative structures

As defined in the literature, e.g.^1–3^, assortative structures correspond to the traditional community structures, in which nodes are more frequently connected to each other inside communities than across them. The disassortative structures do not satisfy this condition. For example, a core-periphery structure^5^ divides the networks nodes into a core part, in which nodes are often the hubs of the networks, and a periphery part with nodes of low-degree connecting to the core nodes. It can be seen as an advantage that the degree-corrected stochastic block model can generate the structures with different mixing patterns. However, when searching for the weak assortative community structures, the inference algorithm using this model may infer the disassortative structure instead^11^.

We generate synthetic networks using the degree-corrected stochastic block model with the parameters *ω*_*rs*_ chosen for the assortative communities as follows

3 $${\omega }_{rs}=\{\begin{array}{cc}\gamma {\omega }_{0} & {\rm{if}}\,r=s,\\ {\omega }_{0} & {\rm{if}}\,r\ne s,\end{array}$$

where a large value of *γ* > 1 results in strong assortative structure while *ω*_0_ controls the sparsity of the network. For the generation of synthetic networks, the degree sequence {*k*_*i*_} is drawn from a power-law distribution with exponent 2.5, and the two blocks are generated with equal size to which the nodes are randomly assigned.

**Figure 1:**
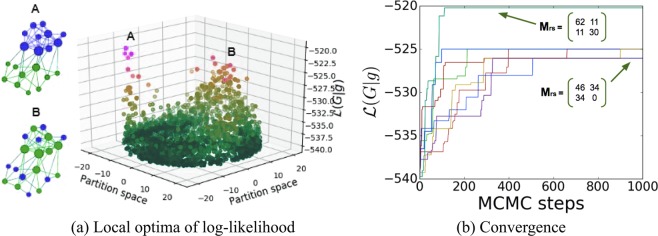
The convergence of 20 Markov chain Monte Carlo (MCMC) trials for the degree-corrected stochastic block model. The local maximum *A* found by MCMC represents the assortative communities, whereas there are other local maxima representing disassortative structures such as point B. (**a**) Multiple locally maximal partitions discovered by the MCMC inference using the degree-corrected stochastic block model; (**b**) Two out of the 20 MCMC trials find the most likely and sought-after assortative partition, while the other trials converge to the local maxima representing disassortative structures. The matrix *M*_*rs*_ indicates the number of edges between any node from block *r* and any node from block *s*, including cases when *r* = *s*.

For the example shown in Fig. 1, given the synthetic networks produced by the degree-corrected stochastic block model, we infer its block assignments {*g*_*i*_} using the Markov chain Monte Carlo (MCMC) algorithm^12,13^. Specifically, the number of communities is set as two for the process of generating it and for recovery of its parameters. To generate the samples, the degree-corrected stochastic block model uses *ω*_0_ = 0.01 and *γ* = 10. Each of the two blocks contains 10 nodes. We scatter the sampled partitions on the x-y plane in Fig. 1(a) while the z-axis indicates the log-likelihood of the corresponding partitions. The details of the MCMC algorithm are provided in the Supplementary Information.

Figure 1 shows the multiple local maxima of the log-likelihood of degree-corrected stochastic block model that exist for our sample network. The inference process finds three local maxima here: (i) partition A which corresponds to the assortative structure matching the ground truth block assignment used for its generation; (ii) partition B which corresponds to a disassortative structure; and (iii) another disassortative partition which is not explicitly marked in the Fig. 1(a). Under the degree-corrected stochastic block model, the MCMC inference starting from random initial partition finds the partition A matching the ground truth block assignment used for its generation in only two out of 20 trials. The other trials converge to the local maxima that represent disassortative structures, as shown in Fig. 1(b).

Since there are multiple local maxima in the log-likelihood of the degree-corrected stochastic block model, the inference algorithm may converge to any of them. In our experiments, the type of the discovered structure depends on the trial starting point and inference algorithm parameters. To avoid such undesired outcomes, we introduce a novel approach applicable to any inference algorithm that we named the Regularized Stochastic Block Model (RSBM). It constrains each node internal degree ratio used in the objective function. This ratio is defined as a fraction of the node’s neighbors that are inside its community. The inference algorithm using the regularized model reliably finds assortative or disassortative structures as directed by the value of a single parameter.

### Regularized stochastic block model

We extend the formulation of the expected number of edges between nodes *i* and *j*, determined by the *Poisson* rate *λ*_*ij*_, in the degree-corrected stochastic block model by redefining it as

4 $${\lambda }_{ij}=(\begin{array}{cc}{\omega }_{{g}_{i},{g}_{j}}{I}_{i}{I}_{j} & {\rm{if}}\,{g}_{i}={g}_{j}\\ {\omega }_{{g}_{i},{g}_{j}}{O}_{i}{O}_{j} & \mathrm{otherwise},\end{array}$$

where any node *l* has two associated parameters *I*_*l*_ and *O*_*l*_. Given Eq. 2, the log-likelihood of generating graph *G* by this regularized stochastic block model can be written as

5 $$ {\mathcal L} (G|g,\omega ,{\bf{I}},{\bf{O}})=2\sum _{i}\,({k}_{i}^{+}\,\log \,{I}_{i}+{k}_{i}^{-}\,\log \,{O}_{i})+\sum _{rs}\,{m}_{rs}\,\log \,{\omega }_{rs}-{\omega }_{rs}{\Lambda }_{rs}$$

where *k*_*i*_^+^ is the number of neighbors of node *i* which are inside the same block given the block assignment *g*, *k*_*i*_^−^ = *k*_*i*_ − *k*_*i*_^+^, and

6 $${\Lambda }_{rs}=(\begin{array}{cc}{(\sum _{i\in r}{I}_{i})}^{2} & {\rm{if}}\,r=s\\ \sum _{i\in r}\,{O}_{i}\sum _{i\in s}\,{O}_{i} & {\rm{if}}\,r\ne s\end{array}$$

To simplify, we write *i* ∈ *r* if *g*_*i*_ = *r*. For block assignment *g*, the maximum-likelihood values of *ω*_*rs*_ are

7 $${\hat{\omega }}_{rs}=\frac{{m}_{rs}}{{\Lambda }_{rs}}\mathrm{.}$$

Dropping the constants and substituting using Eq. 5, we obtain

8 $$ {\mathcal L} (A|g,{\bf{I}},{\bf{O}})=\sum _{rs}\,{m}_{rs}\,\log \,\frac{{m}_{rs}}{{\Lambda }_{rs}}+2\sum _{i}\,({k}_{i}^{+}\,\log \,{I}_{i}+{k}_{i}^{-}\,\log \,{O}_{i})$$

Note that if we set *I*_*i*_ = *O*_*i*_ = 1 here, the log-likelihood above reduces to the definition of standard stochastic block model with Λ_*rs*_ = *n*_*r*_*n*_*s*_ which is exactly the product of the sizes of two blocks *r* and *s*. When *I*_*i*_ = *O*_*i*_ = *k*_*i*_, the second sum on the right hand side (RHS) becomes irrelevant to the maximum likelihood estimation (MLE) result. Hence, the log-likelihood reduces to the definition of degree-corrected stochastic block model in Eq. 8 with Λ_*rs*_ = *κ*_*r*_*κ*_*s*_, i.e., the product of the sums of degrees of nodes in two blocks *r* and *s*. Hence, by introducing here two sets of parameters *I* = {*I*_*i*_} and *O* = {*O*_*i*_} in the edge probability, we obtain a more generalized definition of the stochastic block model.

### Regularization by prior in-degree ratios

In alternative formulation of our model, we define for each node *i* the prior in-degree ratios *f*_*i*_ = *I*_*i*_/(*I*_*i*_ + *O*_*i*_) and *θ*_*i*_ = *I*_*i*_ + *O*_*i*_. By rewriting the second summation on the RHS of Eq. 8, we get

9 $$ {\mathcal L} (G|g,{\bf{I}},{\bf{O}})=\sum _{rs}\,{m}_{rs}\,\log \,\frac{{m}_{rs}}{{\varLambda }_{rs}}-2\sum _{i}\,{k}_{i}H(\frac{{k}_{i}^{+}}{{k}_{i}},{f}_{i})+2\sum _{i}\,{k}_{i}\,\log \,{\theta }_{i}$$

where $H(\frac{{k}_{i}^{+}}{{k}_{i}},{f}_{i})=-\frac{{k}_{i}^{+}}{{k}_{i}}\,\log \,{f}_{i}-\frac{{k}_{i}^{-}}{{k}_{i}}\,\log \,\mathrm{(1}-{f}_{i})$ represents the cross entropy between the observed in-degree ratio $\frac{{k}_{i}^{+}}{{k}_{i}}$ and the prior in-degree ratio *f*_*i*_. By maximizing the log-likelihood defined in Eq. 9, the optimization process tends to find a partition with $\frac{{k}_{i}^{+}}{{k}_{i}}\approx {f}_{i}$ in an effort to reduce the sum of the cross entropy terms. Therefore, the prior in-degree ratios {*f*_*i*_} regularize the observed in-degree ratios $\{\frac{{k}_{i}^{+}}{{k}_{i}}\}$ in the resulting partition.

In the real networks with the traditional community structures, the low degree nodes are generally more likely to have neighbors inside the same block than the high degree nodes are. Suppose the prior in-degree ratio *f*_*i*_ depends only on the degree of node *i*, i.e. *f*_*i*_ = *f*(*k*_*i*_). Then, the function $f(k):{{\mathbb{Z}}}_{+}\to \mathrm{[0,\; 1]}$ should be strictly decreasing. In an assortative partition of the network, we have

• *f*(1) = 1 because a node with degree one must connect to the community it belongs to;

• For *k* ≈ |*V*|, $f(k)\ll 1$ because a super-hub eventually does not belong to any community as its degree is of the order of |*V*|, the number of nodes in the entire network.

A simple function {*f*(*k*)} satisfying this requirement is of the form

10 $$f(k)=\alpha +\frac{(1-\alpha )}{k},$$

where *α* is the only extra parameter we introduce to the regularized stochastic block model (RSBM). Alternatively, we can select a constant *f* ∈ (0, 1) such that

11 $$f(k)=\,\max (f,\frac{1}{k}),$$

where now *f* is the only extra parameter introduced for this form of the function *f*_*i*_. The impact of different choices of *f*_*i*_ on the discovered block assignment is discussed in the following two subsections presenting experimental results.

### Experimental results

We generate the synthetic networks with the assortative communities using the degree-corrected stochastic block model. The parameters *ω*_*rs*_ used in the network generation process are specified by Eq. 3. Selecting a small value of *γ* > 1 in Eq. 3 makes the generated community structures relatively weak, and therefore difficult to detect by the statistical inference using the degree-corrected stochastic block model.

**Figure 2:**
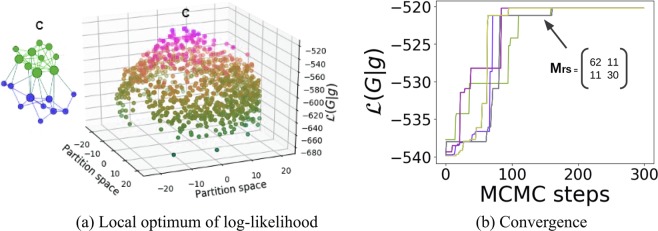
The convergence of 20 Markov chain Monte Carlo (MCMC) trials for the regularized stochastic block model (RSBM) introduced here. All trials converge to the maximal point C which represents an assortative structure. (**a**) One maximal partition observed during the MCMC inference using our model. (**b**) All twenty MCMC trials find the sought-after assortative structure.

We again adapt the Markov chain Monte Carlo (MCMC) algorithm^13^ to infer the block assignments using our proposed regularized stochastic block model. Figure 2(a) shows there is only one unique local maximum, the partition C, found by 20 MCMC trials under the regularized stochastic block model. Therefore, all 20 MCMC trials converge to this unique local maximum. As shown in Fig. 2(b), the inference process finds the correct block assignment in at most 150 MCMC steps. The regularization terms enable the inference to converge to the suitable local maxima.

**Figure 3:**
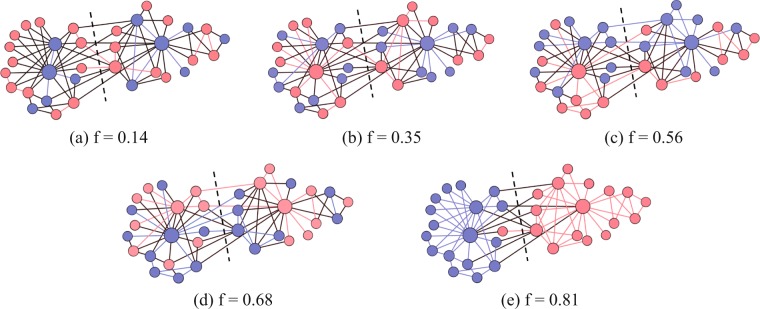
The Karate network partition inferred by Markov chain Monte Carlo under different parameter settings where nodes of the same color belong to the same partition. The black dotted line represents the ground truth partition. A small *f* results in a core-periphery partition of the network while a large *f* leads to an assortative partition. The values of *f* parameter are shown in the corresponding sub-figures captions.

We use the real networks including the Karate club network of Zachary^14^, the Dolphin social network of Lusseau *et al*.^15^ and the network of fictional characters’ interactions in the novel Les Miserables by Victor Hugo^2^ to demonstrate the performance of the regularized model introduced here. The details of each network are presented in the Supplementary Information. For the Karate club network, we evaluate the effect of the regularization terms on the resulting partitions recovered using the Markov chain Monte Carlo (MCMC). For every node *i* in the network, we set the parameter *θ*_*i*_ = *k*_*i*_ and *f*_*i*_ = *max*(*f*, 1/*k*_*i*_) for our regularized stochastic block model. Figure 3 shows the most likely partition of the Karate club network found by MCMC using different *f* values. The color represents the block assignment, and the black dashed line divides the network into two parts in the ground truth partition. As shown in Fig. 3, when *f* = 0.14, the inference algorithm outputs a core-periphery structure which clusters high-degree nodes into the blue block and the remaining low-degree nodes into the red block. This is because the sum of cross entropy terms in Eq. 9 serves as a regularization term which penalizes partitions with the average in-degree ratio much larger than 0.14. As the value of *f* grows, the inference algorithm is becoming more likely to detect assortative structure. When *f* = 0.85, the inferred block assignment matches the ground truth partition of the Karate club network with the exception of one single red node. However, this node has only one connection to each block; thus, it is quite arguable to which block this node should belong.

**Figure 4:**
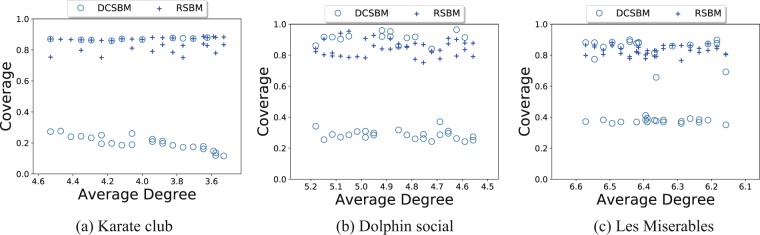
The *coverage* of partitions in the three real networks as a function of the average node degree. The maximal partitions are inferred by the Markov chain Monte Carlo algorithm under the degree-corrected stochastic block model (DCSBM), marked by circles, and its regularized extension (RSBM) introduce here, marked by crosses.

The results in Fig. 4 show that the MCMC inference using our RSBM model on the three real networks finds partitions with higher *coverage* than the ones inferred by the degree-corrected stochastic block model. The *coverage* of a partition^1^ is defined as the ratio of the number of edges with both endpoints in the same block to the total number of edges in the entire network

12 $$\mathrm{coverage}({\bf{g}})=\frac{|\{(i,j)\in E|{g}_{i}={g}_{j}\}|}{|E|}\mathrm{.}$$

A low *coverage* indicates that the resulting partition is disassortative. An ideal assortative partition of the network, where all clusters are disconnected, yields a *coverage* of 1.

Figure 4 shows the *coverages* of the partitions found in the three real networks mentioned above. We randomly remove edges in these networks to further increase their sparsity. The numbers of blocks used in trials for these networks are set to their broadly accepted values in the literature. The results in Fig. 4 indicate that under the degree-corrected stochastic block model (DCSBM) the inference algorithm is likely to miss the assortative structures. It returns instead the disassortative partitions of the network, which also fit the model in such cases. In contrast, the MCMC inference using our regularized stochastic block model (RSBM) almost always produces the assortative structures. Interestingly, the inference using the degree-corrected stochastic block model produces the partitions with the *coverage* values distributed at two levels. This is similar to the case of the synthetic network in Fig. 1 where both the assortative communities and disassortative structures fit the degree-corrected stochastic block model. Which structure is found is determined by random sampling of the potential partitions. In other words, there is no way to guide whether a disassortative or an assortative structure is preferred. Hence, two MCMC trials may return completely different structures. In contrast, the inference using the regularized stochastic block model introduced here and using the prior in-degree ratio *f*_*i*_ = 0.8 + 0.2/*k*_*i*_ is very robust. It only produces partitions with a high *coverage* distributed at the same level of the coverage as the assortative partitions found under the degree-corrected stochastic block model. Moreover, the sparsity of the networks does not have an obvious impact on the resulting partitions under our regularized model.

**Figure 5:**
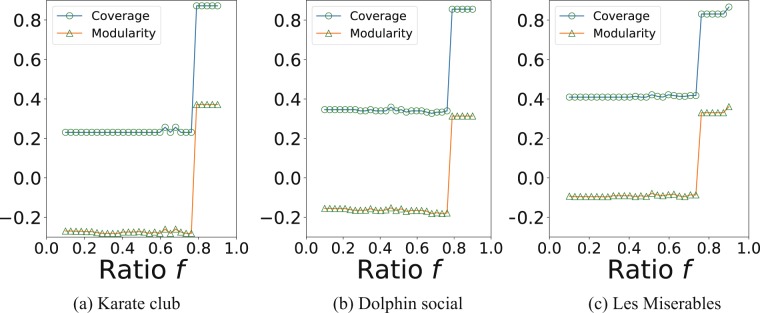
The *coverage* and modularity of partitions in the real networks as functions of parameter *f*. The maximal partitions are inferred by the Markov chain Monte Carlo algorithm under the degree-corrected stochastic block model (DCSBM) and its regularized extension (RSBM) introduced here.

We evaluate the modularity^16^ of resulting partitions of the real networks as a function of parameter *f*. A high modularity indicates the strong community structure. In our experiments, we use a constant *f* such that *f*_*i*_ = *f* for each node *i* in the network with degree *k*_*i*_ > 1/*f*, otherwise *f*_*i*_ = 1/*k*_*i*_. We start with a small *f* value and increase it in each iteration. The MCMC inference uses as a starting point a random partition of the original network initially and then uses the network partition found in the previous iteration. Figure 5 shows that, as the value of *f* increases, in general both the modularity and the *coverage* grow. When *f* is close to 0, the value of *f* does not have any effect on the network partition. However, as *f* becomes larger, then at a certain critical value of about 0.75, a critical transition arises at which the type of the recovered structure changes from disassortative partitions to assortative communities. Using *f* larger than the critical value does not further increase the modularity of the partition. These results indicate that, with one single parameter *f*, the user gains control over the type of structure to which the Markov chain Monte Carlo inference will converge. With a choice of high *f*, the inference algorithm is most likely to detect assortative communities.

## Discussion

The stochastic block model is able to produce a wide variety of network structures, including traditional assortative structures and different from them disassortative structures. In theory, it should be possible to guide the inference algorithm which type of structures is preferred. Moreover, the existence of multiple local maxima of the log-likelihood may cause an inference algorithm to converge to the undesired type of structure. Although there were efforts to enable community detection at different levels of granularity, cf.^17,18^, the need for controlling assertiveness of the solution has not been addressed so far. Here, we apply a simple yet effective constraint on nodes’ internal degree ratios in the objective function. This approach is applicable to any inference algorithm. The resulting algorithm reliably finds assortative or disassortative structure as directed by the value of a single parameter *f*. We validate the model experimentally testing its performance on several real and synthetic networks. The experiments show that the inference using our regularized stochastic block model quickly converges to the sought-after assortative or disassortative structure. In contrast, the inference using the degree-corrected stochastic block model often converges to the local maximal partitions representing the undesired type of structure.

## Methods

### Proving properties of the regularized stochastic block model

Theorem 1 When f_i_ = 1/2 for each node i in the network, the MLE of our RSBM model defined by Eq. 9 becomes the MLE of degree-corrected stochastic block model.

**Figure 6:**
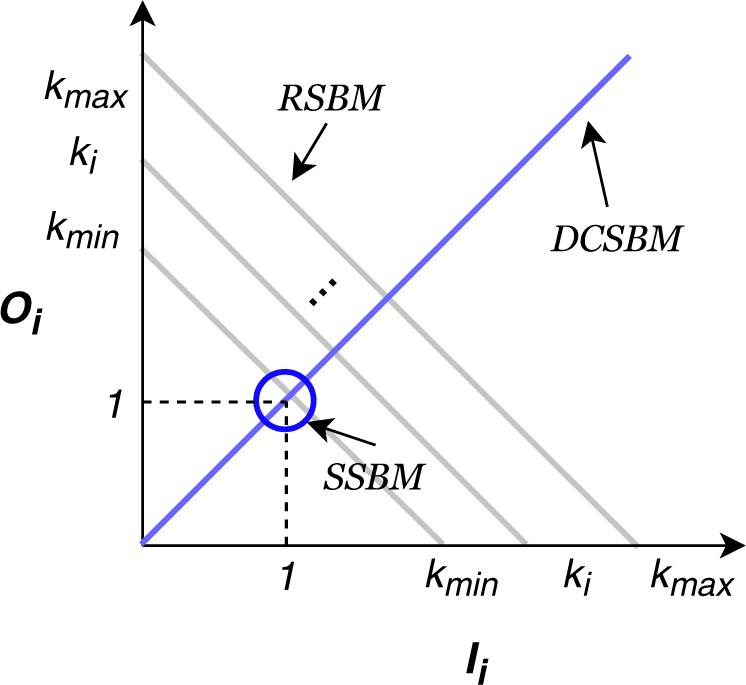
A unified view on the variants of stochastic block models. The standard stochastic block model (SSBM) is represented by the dark blue point (1, 1) since it sets *I*_*i*_ = *O*_*i*_ = 1 for each node *i*. The degree-corrected stochastic block model (DCSBM) requires *I*_*i*_ = *O*_*i*_, which corresponds to the light blue line with a slope *f*_*i*_ = 1/2 in the 2D coordinate plane. The introduced here regularized stochastic block model (RSBM) allows choosing *I*_*i*_ and *O*_*i*_ on the line *I*_*i*_ + *O*_*i*_ = *k*_*i*_ where *k*_*i*_ is the node degree. So our model is represented by a set of points on the parallel gray lines each of which intersects the x and y-axis at integer values corresponding to the degree of a node in the range [*k*_min_, *k*_max_].

According to Theorem 1 (the proofs of Theorem 1, 2 and 3 are provided in the Supplementary Information), if we add a constraint *I*_*i*_ = *O*_*i*_ for every node *i*, the model introduced here reduces to the degree-corrected stochastic block. Figure 6 illustrates the relationship between the most commonly used variants of stochastic block models. If we plot the constraints of these models on a 2D plane with *I*_*i*_ and *O*_*i*_ as the *x* and *y* axes, respectively, the standard stochastic block model (SSBM) sets *I*_*i*_ = *O*_*i*_ = 1 for each *i*, which maps to the point (1, 1) in the 2D coordinate plane. The constraint *I*_*i*_ = *O*_*i*_ which represents the degree-corrected stochastic block model (DCSBM) maps onto the line with a slope *f*_*i*_ = 1/2 in the 2D coordinate plane. Here, we extend the stochastic block model in two different directions: (i) using a constraint on *θ*_*i*_ = *I*_*i*_ + *O*_*i*_; (ii) choosing *I*_*i*_ and *O*_*i*_ on the line with a customized slope *f*_*i*_. It turns out the latter, like the degree-corrected stochastic block model, preserves the degree sequence of the network.

Theorem 2 Given any customized {f_i_} and the corresponding maximum-likelihood estimator ${\hat{\theta }}_{i}$, our model defined by Eq. 9 preserves the degree sequence of a network, so the expected degree of node i generated by the RSBM model with ${\hat{\theta }}_{i}$ is

13 $$\sum _{j}{\lambda }_{ij}={k}_{i}\mathrm{.}$$

### Information-theoretic interpretations

While the maximum likelihood estimator ${\hat{\theta }}_{i}$ preserves the degree sequence in the observed network, we find that the closed-form analytic expression of such estimator is hard to obtain. However, when we impose constraints that for each *i*, *θ*_*i*_ = *k*_*i*_ and look for the MLE of *f*_*i*_ for every *i*, the log-likelihood of the model introduced here has an interesting interpretation uncovered by the following theorem.

Theorem 3 When θ_i_ = k_i_ for every node i, maximizing the log-likelihood of Eq. 9 is equivalent to maximizing the target function

14 $$ {\mathcal L} ={{\mathbb{D}}}_{KL}({p}_{{\rm{degree}}}(r,s)||{p}_{{\rm{null}}}(r,s))-2{{\mathbb{E}}}_{{k}_{i}}[H(\frac{{k}_{i}^{+}}{{k}_{i}})]$$

where the first term represents the Kullback-Leibler (KL) divergence between the edge distribution under the block model and the corresponding distribution under the null model which randomizes the edges inside and across blocks respectively, while the second term is twice the expectation of the binary entropy of in-degree ratio *k*_*i*_^+^/*k*_*i*_.

Imposing the constraint *I*_*i*_ + *O*_*i*_ = *k*_*i*_, we obtain a new model which involves a regularization term of the expected entropy of in-degree ratio *k*_*i*_^+^/*k*_*i*_. According to Theorem 3, maximizing the log-likelihood of this model has a physical interpretation: the MLE of the RSBM model increases the KL divergence between the edge distribution under the block model and the corresponding edge distribution under the *null* model, and, at the same time, it decreases the expected entropy of the in-degree ratio *k*_*i*_^+^/*k*_*i*_. Intuitively, the *null* model here separates the edges inside a block and the edges across different blocks. It assumes that all edges inside blocks are statistically equivalent, and so are all edges across different blocks. In contrast, the *null* model of the degree-corrected stochastic block model^8^ mixes all the edges, assuming all the edges are statistically equivalent - the KL divergence term in its *null* model does not distinguish the edges inside the same block and those across different blocks. On the other hand, the sum of entropy function *H*(*k*_*i*_^+^/*k*_*i*_) controls the identification of the edges’ types. In Eq. 14, *H*(*k*_*i*_^+^/*k*_*i*_) is the entropy function which achieves its minimum when either *k*_*i*_^+^/*k*_*i*_ → 0 or *k*_*i*_^+^/*k*_*i*_ → 1. Therefore, the MLE of this model is likely to classify the edges of a node as either all in-edges or all out-edges – the former case is likely to detect assortative communities and the latter case infers disassortative structure as the most probable block assignment.

For community detection problems, it is rarely observed that all neighbors of a node are not located in the same block, especially for nodes with low degrees. Unlike traditional community detection algorithms, the definitions of stochastic block model and its mentioned above variants do not explicitly control the fraction of neighbors in different blocks. Instead, they rely on the specific statistical inference to determine the block assignments. This observation inspires us to regularize the traditional stochastic block model on the in-degree ratio. Specifically, our Regularized Stochastic Block Model (RSBM) introduced here maximizes the objective function of Eq. 9 with *θ*_*i*_ = *k*_*i*_ and *f*_*i*_ defined by the prior in-degree ratio. As explained above, the KL divergence in RSBM plays the same role as the divergence does in the degree-corrected stochastic block model. The expected cross-entropy term serves as a regularization term to control the resulting partition. Intuitively, when *f*_*i*_ is close to one, then the inference algorithm tends to cluster nodes in a network into dense modules. Otherwise, when *f*_*i*_ is close to zero, then disassortative partitions such as the core-periphery structure are likely to be discovered because the regularized block model tends to assign adjacent nodes into different blocks to decrease *k*_*i*_^+^.

## Supplementary information

Supplementary Discussion, Proofs of Theorems, and Description of Experiments

## Data Availability

The network data sets used for the evaluation of our proposed model are available at http://konect.uni-koblenz.de/networks/.
