# Supplementary material for: A Regularized Stochastic Block Model for the robust community detection in complex networks
Source: Sci Rep. 2019 Sep 13;9:13247. doi: 10.1038/s41598-019-49580-5 (PMC6744415; doi:10.1038/s41598-019-49580-5)
Supplement: Supplementary file 1 — Supplementary Discussion, Proofs of Theorems, and Description of Experiments [file 41598_2019_49580_MOESM1_ESM.pdf]

Supplementary Discussion, Proofs of Theorems, and Description of Experiments

Xiaoyan Lu[1], Boleslaw K. Szymanski[1,2*]
[1] Social and Cognitive Networks Academic Research Center and Department of Computer Science, Rensselaer Polytechnic Institute, Troy NY 12180, USA
[2] Społeczna Akademia Nauk, Łódź, Poland

Supplementary Discussion

Review of the degree-corrected stochastic block model

Since the standard stochastic block model makes nodes in the same block statistically indistinguishable, the most likely block assignment often places the nodes of similar degrees in a block, resulting in blocks of nodes with homogeneous degrees, too limited to represent the traditional community structures.

The degree-corrected stochastic block model [1] clusters together the nodes with heterogeneous degrees and defines the expected number of edges between nodes i and j as $\lambda_{ij} = \omega_{g_i,g_j}\beta_i\beta_j$ where for an arbitrary node l, its block assignment is denoted g_l and its model parameter is denoted β_l. This simple yet effective extension improves the performance of the models for statistical inference of a block structure in the real-world networks because nodes in a community tend to have broad degree distributions. To simplify technical derivations, authors in [1] approximate the *Bernoulli* distribution with the *Poisson*. This approximation is tight when the product of the number of nodes n and edge probability p is small. Given a partition of the network, i.e., its block assignments $\{g_i\}$, the authors of [1] obtain the maximum likelihood estimates of the model parameters equal to $\hat{\beta}_i = \frac{k_i}{\kappa_{g_i}}$ and $\hat{\omega}_{rs} = m_{rs}$, where k_i is the degree of node i, κ_r is the sum of the degrees of all nodes in a block r, and m_{rs} is the total number of edges between blocks r and s, so if $r = s$, it is twice the number of edges in r. Note that the self-loop edges and multi-edges are allowed in this model. Ignoring the constant terms which are irrelevant to the statistical inference of $\mathbf{g}$, the log-likelihood of a graph G given the partition $\mathbf{g}$ can be expressed as

$$\mathcal{L}(G|\mathbf{g}) = \sum_{rs} m_{rs} \log \frac{m_{rs}}{\kappa_r \kappa_s}, \tag{1}$$

This criterion has an information-theoretic interpretation when it is presented in the alternative form

$$\mathcal{L}(G|\mathbf{g}) = \sum_{rs} \frac{m_{rs}}{2m} \log \frac{m_{rs}/2m}{(\kappa_r/2m)(\kappa_s/2m)}, \tag{2}$$

which shows that this log-likelihood represents the KullbackLeibler divergence between the probability of observing an edge (i,j) between block r and s in real network, i.e., $P[i \in r, j \in s] = m_{rs}/2m$, and the probability of observing

*E-mail: szymab@rpi.edu

such edge in a random graph with the same degree sequence, i.e., $P'[i \in r, j \in s] = (\kappa_r/2m)(\kappa_s/2m)$. Seen this way, the statistical inference maximizes the information gain when replacing the generative model of a random graph model with the degree-corrected stochastic block model. In other words, the mostly likely partition is the one that requires the most information to describe the observed graph starting from a random graph that does not have block structure at all.

It is worth mentioning that, in the standard stochastic block model where the number of edges is drawn from the *Bernoulli* distribution, it is rare that ω_{rs} is close to 1. This is because the numbers of the edges between blocks are usually very small when the network is sparse. A *Bernoulli* random variable with a small mean is well approximated by a *Poisson* random variable having the same mean [2]. For this reason, [3] found no significant difference in the solutions produced by the model described here that uses the *Poisson* distribution instead of the *Bernoulli* distribution used in the original model.

Markov chain Monte Carlo

We use the Markov chain Monte Carlo (MCMC) algorithm [4] to infer the block assignment using the stochastic block model and its extensions. The MCMC algorithm samples the distribution of all possible block assignments in the network partitions space. Suppose there are K blocks and N nodes in a network. The naive Markov chain Monte Carlo approach is not practical because the size of the partition space $O(N^K)$ which grows exponentially in the number of blocks K. Therefore, Peixoto [5] proposes the optimized MCMC algorithm with the greedy heuristic to infer the block assignment. Initially, every node in the network is assigned to one random block independently. Then, one attempts to move a node from block r to s with a probability conditioned on its neighbor's block assignment t. The proposal probability function is defined as

$$p(r \to s|t) = \frac{m_{ts} + \epsilon}{\sum_s m_{ts} + \epsilon K} \tag{3}$$

where $\epsilon > 0$ is a free parameter to fulfill the ergodicity condition which requires that any block assignment can be reached from the current block assignment in the finite and aperiodic expected number of steps. When $\epsilon \to \infty$, the proposal function reduces to the naive scheme which assigns a random block to the current node. However, in such case, the possibility of current node being assigned to the correct block assignment is very low, thus, majority of the moves do not increase the log-likelihood. Consequently, these moves are rejected very frequently, wasting the computational resources. Applying a relatively small ϵ makes the moves more likely to get accepted. This is because if there are a lot of edges between blocks s and t, a node with many neighbors in block t is likely to reside in block s. Consequently, moving node from block s to t is more likely to be accepted, saving the computational cost of many rejected proposals.

To ensure the detailed balance, each proposed assignment is accepted with a probability a in the Metropolis-Hastings fashion [6] given by

$$a = \min\left\{\exp(\Delta\mathcal{L})\frac{\sum_t n_t p(s \to r|t)}{\sum_t n_t p(r \to s|t)}\right\}, \tag{4}$$

where the node of the proposed assignment has n_t neighbors in block t and $\Delta\mathcal{L}$ is the change of log-likelihood after each move. A list of edges which are adjacent to each block are used to compute Eq. 4, which can be done in $O(k_i)$ time [5] where k_i is the degree of node i. Thus, processing moves for all nodes in the network requires $O(E)$ operations where E is the number of edges in the networks.

Supplementary Proofs of Theorems

Proof of Theorem 1

Proof. If $f_i = 1/2$ for each node i, then $I_i = O_i = \theta_i/2$ and $\sum_{i\in r} I_i = \sum_{i\in r} O_i = \sum_{i\in r} \theta_i/2$. Hence, $\Lambda_{rs} = \Theta_r/2 \times \Theta_s/2$ where $\Theta_r = \sum_{i\in r}\theta_i$. Also, it is obvious that the sum of cross entropy on the RHS of Eq. 9 is a constant term given the block assignment $\mathbf{g}$. The log-likelihood of our model defined by Eq. 9 can be rewritten in the form

$$\mathcal{L}(G|\mathbf{g},\mathbf{I},\mathbf{O}) = \sum_{rs} m_{rs} \log \frac{m_{rs}}{\Theta_r \times \Theta_s} + 2\sum_i k_i \log\theta_i + const.$$

where all the constant terms are grouped into the last term on the RHS. Setting the derivative of the RHS over θ_i to zero, we obtain $k_i/\theta_i = \sum_s m_{g_i,s}/\Theta_{g_i}$ which means that for every node i the MLE estimator $\hat{\theta}_i = \Theta_{g_i} k_i/\kappa_{g_i}$. Plugging $\hat{\theta}_i$ into the log-likelihood above, we obtain the log-likelihood expression in Eq. 1 after dropping all the constant terms. □

Proof of Theorem 2

Proof. For given $\{f_i\}$, the derivative of the log-likelihood defined in Eq. 8 over θ_i is zero when the maximum likelihood estimator $\hat{\theta}_i$ satisfies the condition

$$\frac{k_i}{\hat{\theta}_i} = \frac{m_{rr}}{\sum_{i\in r} f_i\hat{\theta}_i} f_i + \frac{\sum_{s:s\neq r} m_{rs}}{\sum_{i\in r}(1-f_i)\hat{\theta}_i}(1-f_i) \tag{5}$$

Even without the closed-form expression of $\hat{\theta}_i$, Eq. 5 ensures that the expected degree of node i is

$$\sum_j \lambda_{ij} = I_i \sum_{j\in g_i} I_j \frac{m_{g_i,g_i}}{\lambda_{g_i,g_i}} + O_i \sum_{j\notin g_i} O_j \frac{m_{g_i,g_j}}{\lambda_{g_i,g_j}} \tag{6}$$

$$= I_i \frac{m_{g_i,g_i}}{\sum_{j\in g_i} I_j} + O_i \frac{\sum_{b:b\neq g_i} m_{g_i,b}}{\sum_{j\in g_i} O_j} \tag{7}$$

Since $\theta_i = \hat{\theta}_i$, we have $I_i = f_i\hat{\theta}_i$ and $O_i = (1-f_i)\hat{\theta}_i$, so the RHS of Eq. 7 is actually the RHS of Eq. 5 multiplied by $\hat{\theta}_i$, hence it is equal to observed node degree k_i in the network. Therefore, $\sum_j \lambda_{ij} = k_i$. □

Proof of Theorem 3

Proof. We start with the equivalent definition of the generalized model in Eq. 8. The first-order derivatives of the log-likelihood over I_i and O_i are

$$\frac{\partial \mathcal{L}}{\partial I_i} = -m_{g_i g_i} \frac{2\sum_{j\in g_i} I_j}{(\sum_{j\in g_i} I_j)^2} + \frac{2k_i^+}{I_i} \tag{8}$$

$$\frac{\partial \mathcal{L}}{\partial O_i} = -\sum_{s:s\neq g_i} m_{g_i s} \frac{2\sum_{i\in s} O_i}{\sum_{j\in g_i} O_j \cdot \sum_{i\in s} O_i} + \frac{2k_i^-}{O_i} \tag{9}$$

Given the block assignment $\mathbf{g}$, when log-likelihood achieves its maximum, the stable points of I_i and O_i satisfy $\frac{\partial \mathcal{L}}{\partial I_i} = \frac{\partial \mathcal{L}}{\partial O_i} = 0$, which leads to the equations

$$\forall i \in r: \qquad \frac{k_i^+}{I_i}\sum_{i\in r} I_i + \frac{k_i^-}{O_i}\sum_{i\in r} O_i = \sum_{i\in r} I_i + O_i = \kappa_r \tag{10}$$

Suppose there are R nodes in block r, so we have R independent equations shown above and the other R equations are $I_i + O_i = \theta_i = k_i$ which represent the assumed constraints. These $2R$ independent equations produce at most one unique set of solutions for the $2R$ variables. It is obvious that $I_i = k_i^+$ and $O_i = k_i^-$ satisfy all these $2R$ equations. Therefore, Eq. 9 can be rewritten as

$$\mathcal{L} = \sum_{rs} \frac{m_{rs}}{2m} \log \frac{m_{rs}/2m}{\Lambda_{rs}/(2m)^2} - 2\sum_i \frac{k_i}{2m} H\left(\frac{k_i^+}{k_i}\right) \tag{11}$$

where $\Lambda_{rs} = \kappa_r^+ \kappa_r^+$ if $r = s$; $\Lambda_{rs} = \kappa_r^- \kappa_s^-$ otherwise. Here $\kappa_r^{+/-} = \sum_{i\in r} k_i^{+/-}$ are the sum of internal and external degrees of nodes respectively.

The first sum on the RHS of above equation represents the KullbackLeibler divergence between the distribution of edges residing across blocks r and s, i.e., $p_{\text{degree}}(g_i = r, g_j = s) = \frac{m_{rs}}{2m}$ and the corresponding *null* distribution is defined as

$$p_{\text{null}}(g_i = r, g_j = s) = \begin{cases} \frac{\kappa_r^-}{2m}\frac{\kappa_s^-}{2m} & \text{if} \quad r \neq s \\ \frac{\kappa_r^+}{2m}\frac{\kappa_s^+}{2m} & \text{if} \quad r = s \end{cases} \tag{12}$$

Note that the *null* model here does not require that all the edges across the entire network are randomly distributed. But it does require that, the edges inside each block and across different blocks are so distributed.

Since $\sum_i k_i = 2m$, the term $\frac{k_i}{2m}$ can be treated as the probability of selecting an edge of node i. Thus, the second sum on the RHS can be treated as the expectation of binary entropy function $H\left(\frac{k_i^+}{k_i}\right)$, i.e., $\mathbb{E}_{k_i}\left[H\left(\frac{k_i^+}{k_i}\right)\right] = \sum_i \frac{k_i}{2m} H\left(\frac{k_i^+}{k_i}\right)$. □

Supplementary Description of Experiments

Real networks for experiments

Supplementary Table S1 shows the number of nodes and edges of the real networks used for experiments. The number of blocks is set to their values generally

accepted in the previous publications. As shown in Supplementary Table S1, the Karate club network, the Dolphin social network and the Les Miserables characters network are relatively sparse because their average degrees range from 4 to 6. To investigate the performance of the regularized model, we add “noises” to these graph by randomly removing their edges, further increasing the sparsity of these networks. For each network, we iteratively remove random edges from the original set of edges and repeat this process twice (each time starting from the same original network). If the removal of an edge results in the isolated nodes in the network, we remove them.

Supplementary Table S1: Real networks for experiments. The number of blocks for each network is set to its value broadly accepted in the previous publications.

Network	#Nodes	#Edges	#Blocks	Ref.
Karate club	34	78	2	[7]
Dolphin social network	62	159	2	[8]
Characters from Les Miserables	77	254	6	[9]

Dimensionality reduction of the partition space

In Figures 1 and 2, we project the multidimensional space of partitions into the 2D x-y plane. Specifically, for each MCMC trial, we record all the sampled partitions of the network and their corresponding log-likelihoods. Given two sampled partitions $\{g_i\}$ and $\{b_i\}$, a new partition $\{l_i\}$ is randomly generated such that $l_i = g_i$ with probability 0.5 and $l_i = b_i$ otherwise. Then, we scatter all the sampled partitions along with the randomly generated partitions on the x-y plane of Figure 1. In this figure, the multi-dimensional partition space is projected to a two-dimensional x-y plane by the multidimensional scaling (MDS) algorithm [10]. The z-axis indicates the log-likelihood of the partitions.

Illustrative toy networks

We use illustrative toy network to demonstrate the effect of regularization introduced here on the stochastic block model. Consider the “twin stars” network as shown in Supplementary Figure S1.

Supplementary Table S2: The log-likelihoods of the three partitions of the twin stars network under the standard stochastic block model (SSBM), the degree-corrected stochastic block model (DCSBM) and our block model with regularization (RSBM). The partitions are shown in Supplementary Figure S1 and the corresponding maximum log-likelihood values are marked by the bold font.

Model	Core-periphery	Twisted core-periphery	Assortative community
SSBM	**-12.47664**	-18.38972	-24.66870
DCSBM	-44.66540	**-39.55004**	-45.82902
RSBM ($\alpha = 0.3$)	-20385.51476	-377.52624	**-44.68795**
RSBM ($\alpha = 0.6$)	-20394.46861	-368.57239	**-43.38465**
RSBM ($\alpha = 0.9$)	-20416.64932	-346.39168	**-43.35718**

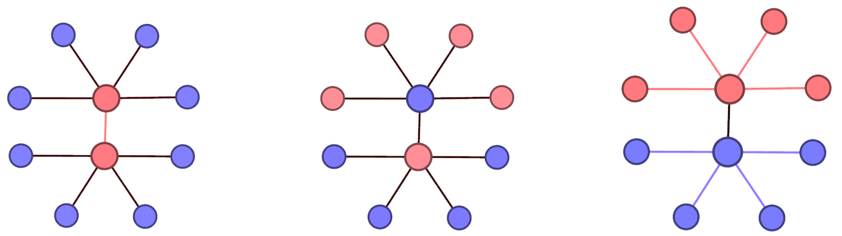

(a) Core-periphery (b) Twisted Core-periphery (c) Assortative Community

Supplementary Figure S1: The twin stars network and its three possible partitions. The color represents a node's block assignment. **(a)** Core-periphery partition with coverage 0.11; **(b)** Twisted core-periphery partition with coverage 0; **(c)** Assortative community partition with coverage 0.89.

There are three possible partitions of this twin stars network. The first option is to cluster the two central hubs into one block and the remaining nodes into another block. This structure corresponds to a core-periphery structure as shown in Supplementary Figure S1(a) where the color of a node represents its block assignment. If we exchange the block assignments of the nodes connected to the two hubs, we obtain a twisted core-periphery structure as shown in Supplementary Figure S1(b). Both of these two partitions are disassortative. For community detection problems, however, the most suitable partition should divide the network into two identical stars, which corresponds to the partition shown in Supplementary Figure S1(c).

Supplementary Table S2 lists the log-likelihoods of these three partitions under the standard stochastic block model (SSBM), the degree-corrected stochastic block model (DCSBM) and our block model with regularization terms (RSBM). Under the degree-corrected stochastic block model, the log-likelihood of the assortative community partition is smaller than the log-likelihood of the inappropriate twisted core-periphery partition, whereas our new model manages to identify the assortative community partition as the most appropriate one. We tested the log-likelihood of the regularized stochastic block model with the $f(k)$ function as defined by Eq. 10 using the model parameter $\alpha = 0.3$, 0.6 and 0.9, respectively. Note that, in Eq. 9, it is possible that $\Lambda_{rs} = 0$ if a block r includes only degree-one nodes as $f(1) = 1$, causing a log-likelihood of negative infinity according to the model definition. In this case, we set the term $m_{rs} \log m_{rs}/\Lambda_{rs} = -10,000$ in Eq. 9 to ensure the log-likelihood is small enough. In general, larger α values would result in a larger gap in the log-likelihoods of the assortative community partition and the other two partitions. However, all the possible α values $0.3, 0.6$ and 0.9 ensure that the assortative community partition fits the regularized model introduced here much better than the other candidates. The reason is that, no matter what value of α gets selected here, we have $f(1) = 1$. To reduce the cross entropy terms in Eq. 14, the inference algorithm is likely to assign a degree-one node to the block in which its sole neighbor resides. Therefore, the inference algorithm is likely to return the assortative communities as the most likely partition of the network.

References

[1] Karrer, B. & Newman, M. E. J. Stochastic blockmodels and community structure in networks. *Phys. Rev. E.* **83**(1), 016107 (2011).

[2] Perry, P. & Wolfe, P. Null models for network data. *arXiv*, 1201.5871 (2012).

[3] Zhao, Y., Levina, E. & Zhu, J. Consistency of community detection in networks under degree-corrected stochastic block models. *Ann. Statist.*. **40**(4), 2266–2292 (2012).

[4] Nasrabadi, N. Pattern recognition and machine learning. *J. Elect. Imag.*. **16**(4), 049901 (2007).

[5] Peixoto, T. Efficient Monte Carlo and greedy heuristic for the inference of stochastic block models. *Phys. Rev. E.* **89**(1), 012804 (2014).

[6] Metropolis, N., Rosenbluth, A., Rosenbluth, M., Teller, A. & Teller, E. Equation of state calculations by fast computing machines. *J. Chem. Phys.*. **21**(6), 1087–1092 (1953).

[7] Zachary, W. An information flow model for conflict and fission in small groups. *J. Anthro. Res.*. **33**(4), 452–473 (1977).

[8] Lusseau, D., Schneider, K., Boisseau, O., Haase, P., Slooten, E. & Dawson, S. The bottlenose dolphin community of Doubtful Sound features a large proportion of long-lasting associations. *Behav. Ecol. Soc.*. **54**(4), 396–405 (2003).

[9] Newman, M. E. J. & Girvan, M. Finding and evaluating community structure in networks. *Phys. Rev. E.* **69**(2), 026113 (2004).

[10] Borg, I., Groenen, P. & Mair, P. Applied Multidimensional Scaling and Unfolding. (Springer, 2017).
